# Neural Tracking of Speech Acoustics in Noise Is Coupled with Lexical Predictability as Estimated by Large Language Models

**DOI:** 10.1523/ENEURO.0507-23.2024

**Published:** 2024-08-14

**Authors:** Paul Iverson, Jieun Song

**Affiliations:** ^1^Department of Speech, Hearing and Phonetic Sciences, University College London, London WC1N 1PF, United Kingdom; ^2^School of Digital Humanities and Computational Social Sciences, Korea Advanced Institute of Science and Technology, Daejeon 34141, Republic of Korea

**Keywords:** auditory tracking, lexical processing, second language, speech in noise

## Abstract

Adults heard recordings of two spatially separated speakers reading newspaper and magazine articles. They were asked to listen to one of them and ignore the other, and EEG was recorded to assess their neural processing. Machine learning extracted neural sources that tracked the target and distractor speakers at three levels: the acoustic envelope of speech (delta- and theta-band modulations), lexical frequency for individual words, and the contextual predictability of individual words estimated by GPT-4 and earlier lexical models. To provide a broader view of speech perception, half of the subjects completed a simultaneous visual task, and the listeners included both native and non-native English speakers. Distinct neural components were extracted for these levels of auditory and lexical processing, demonstrating that native English speakers had greater target–distractor separation compared with non-native English speakers on most measures, and that lexical processing was reduced by the visual task. Moreover, there was a novel interaction of lexical predictability and frequency with auditory processing; acoustic tracking was stronger for lexically harder words, suggesting that people listened harder to the acoustics when needed for lexical selection. This demonstrates that speech perception is not simply a feedforward process from acoustic processing to the lexicon. Rather, the adaptable context-sensitive processing long known to occur at a lexical level has broader consequences for perception, coupling with the acoustic tracking of individual speakers in noise.

## Significance Statement

In challenging listening conditions, people use focused attention to help understand individual talkers and ignore others, which changes their neural processing for speech at auditory through lexical levels. However, lexical processing for natural materials (e.g., conversations, audiobooks) has been difficult to measure, because of limitations of tools to estimate the predictability of individual words in longer discourses. The present investigation uses a contemporary large language model, GPT-4, to estimate word predictability, and demonstrates that listeners make online adaptations to their auditory neural processing in accord with these predictions; neural activity more closely tracks the acoustics of the target talker when words are less predictable from the context.

## Introduction

Speech is often understood under challenging conditions (e.g., noise, unfamiliar accents, distractions), and to some extent we can use focused attention (i.e., listening effort) to adjust our perceptual and cognitive processes to these circumstances (Pichora-Fuller et al., 2016). Real-world adaptations like these can be better assessed under naturalistic listening conditions rather than having subjects judge isolated experimental stimuli (Hamilton and Huth, 2020). For example, EEG can be measured while listeners are attending to connected speech (e.g., audiobooks), and machine learning can be applied across a range of time lags to find neural signals that track speech acoustics (Ding and Simon, 2012, 2014; Crosse et al., 2016, 2021; Obleser and Kayser, 2019; Brodbeck and Simon, 2020). These methods have found that listeners can enhance their auditory neural tracking for attended talkers over ignored distractors (Kerlin et al., 2010; Ding and Simon, 2012).

The same methodologies can be applied to understand how the brain tracks the lexical content of speech, but such investigations require an accurate assessment of the lexical–semantic relationships between individual words and their previous context (Ding et al., 2016; Broderick et al., 2018), which is more difficult in natural materials than in traditional sentence corpora that control predictability (Kutas and Federmeier, 2000; Federmeier, 2007; Strauss et al., 2013). Lexical modeling is an area undergoing rapid advancement, and here we use a contemporary large language model (i.e., GPT-4 Chat Completions API, released 6 July 2023) to assess the predictability of individual words. Until recently, lexical predictability has been mostly calculated using the cooccurrence of short word strings in text databases (*n*-grams) or by the semantic similarity between words and their immediate context (Broderick et al., 2018, 2021; Koskinen et al., 2020; Gillis et al., 2021). Advances in large language models allow us to examine predictability over a larger scale, as a broader and more pervasive factor in speech understanding (Weissbart et al., 2020; Heilbron et al., 2022).

Of particular interest is whether lexical expectations affect auditory processing. We know that top-down attention (e.g., choosing to attend to a talker and ignore others) has effects on early auditory cortical processing (Kerlin et al., 2010; Ding and Simon, 2012) and that lexical predictability promotes speech in noise performance (Miller et al., 1951). However, it is a matter of long-standing debate within the word recognition literature whether lexical processing feeds back to affect lower levels or is entirely a feedforward process (Norris et al., 2000; Magnuson et al., 2003; McClelland et al., 2006; Norris and McQueen, 2008). EEG work has found that auditory tracking tends to be stronger when speech is more intelligible (Howard and Poeppel, 2010; Gross et al., 2013; Peelle et al., 2013; Ding and Simon, 2014; Etard and Reichenbach, 2019) and that the semantic similarity of a word with the previous context can promote auditory tracking, at least for clear speech without the presence of noise (Broderick et al., 2019). In the present study, we contribute to the evidence by assessing whether contemporary lexical predictions are linked to modulations in the tracking of speech acoustics in the presence of a competing talker.

Listeners attended to one talker while ignoring another, and EEG was recorded using a 64-channel active electrode system. The materials were read newspaper and magazine articles, spoken by two female native speakers of southern British English, that were presented at the same amplitude but with a simulated 45° spatial separation between talkers to reduce auditory masking effects (Song et al., 2020). We varied the task and listeners to obtain a wider view of our acoustic and lexical measures. All listeners were asked to listen to the target articles and were given short comprehension questions to promote compliance; half of these listeners simultaneously performed a visual *n*-back task to assess the role of divided attention. In addition, our listeners were native and non-native (Spanish) speakers of English, a factor that shown previously to affect both auditory and lexical processing (Song and Iverson, 2018; Song et al., 2020).

## Materials and Methods

### Subjects

The listeners were 28 native speakers of southern British English and 28 native speakers of Spanish, 30 female and 26 male. All listeners communicated in English in their daily life and were living in London at the time of test. They were 18–40 years old and had no self-reported hearing or language disorders. Three listeners were omitted from the analysis because of EEG recording difficulties (corrupted trigger values).

### Materials and testing procedure

We used 16 newspaper and magazine articles drawn from a range of sources (i.e., Evening Standard, BBC, Aeon, The New Yorker, Boston Review, Architectural Record, The Industrial Archaeology News, Serious Eats). Two female speakers of southern British English read eight articles each, with each recording edited to be ∼4 min in duration, with disfluencies and repetitions removed. There were an average of 74 pauses within each story (12% of words), with an average pause duration of 0.69 s. Eight stimuli were created by mixing the recordings from two speakers, equated in terms of RMS amplitude. The attended speaker was counterbalanced between subjects (i.e., half of listeners attended to one speaker and half attended to the other). The targets and maskers were processed with head-related transfer functions that reproduced the acoustic effects of presenting sound at different spatial locations (Algazi et al., 2001). The target talker was always presented at 0° (front of the head), and the distractor was 45° to the left, with the stimuli presented using insert headphones (Etymotic ER-1) at 67 dB SPL.

**Figure 1.**
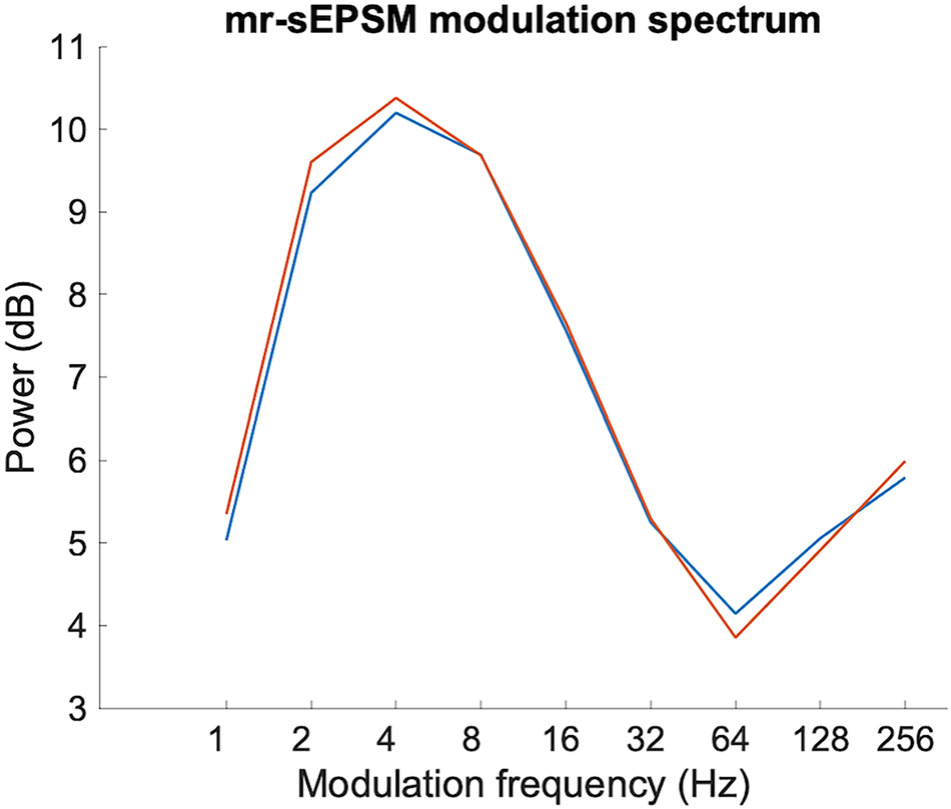
Modulation spectra for the two speakers in this study, demonstrating a close match between talkers with a modulation peak at 4 Hz.

For reference, Figure 1 displays the modulation spectra of the two talkers, calculated according to the mr-sEPSM multiresolution speech-based envelope power spectrum model (Jorgensen et al., 2013). The speakers were very well matched, with a peak near 4 Hz (i.e., at the boundary between delta and theta ranges for EEG) and a secondary peak toward the F0 of the speech (median F0 was 203 and 204 Hz for the two speakers).

After the presentation of each recording, listeners were given two comprehension questions about the target article, both testing memory for a specific fact in the article, in order to encourage compliance with the listening task instructions. The stimuli were presented in a random order for each subject, and subjects were allowed to take breaks between blocks.

A visual *n*-back task (Kirchner, 1958) was performed by half of the subjects (i.e., balanced across language background and target talker). Subjects saw, one at a time in a random order, eight abstract images designed to resemble nonexistent corporate logos; they did not contain text. The images were presented with a jittered duration (0.25–0.55 s), and 25% of the images were a repetition of the image that appeared immediately before. Subjects saw these images on a screen and pressed a button when they recognized that there was a 1-back image repetition.

### EEG processing

EEG was recorded using a Biosemi Active Two System with 64 electrodes and a sampling rate of 2,048 Hz. Electrode impedances were kept below 25 kΩ. Preprocessing was performed in Matlab. The recordings were referenced to the left and right mastoids, high-pass filtered at 0.1 Hz with a zero-phase first-order Butterworth filter, and rereferenced to an artifact-rejected average (de Cheveigne and Arzounian, 2018). Blinks and eye movement artifacts were projected out using denoising source separation (de Cheveigne and Simon, 2008). The electrode PO7 was dropped for all subjects because it was consistently noisy.

For mTRF analysis, the recordings were down sampled to 32 Hz, high-pass filtered with a zero-phase first-order Butterworth filter at 0.1 Hz, low-pass filtered with a zero-phase first-order Butterworth filter at 8 Hz, divided into separate recording blocks for each article (i.e., 8 blocks per subject), and normalized. We used version 2.3 of the mTRF toolbox (Crosse et al., 2016). A leave-one-out cross-validation procedure was used, such that the neural components for each block were calculated based on components trained on the other seven blocks, with a separate cross-validated procedure fitting optimum lambda values within the training blocks (i.e., fit by the mTRFcrossval function in the mTRF toolbox, with a range from 10^−3^ to 10^12^). A decoder model was used with lags between −250 and 750 ms, mapping the 63 channels of EEG back to the stimulus functions. Statistical analyses were conducted on the backward-projected neural data (i.e., correlations between stimulus functions and neural components). The coefficients were then forward projected to allow for better interpretation of the neural sources; the raw decoding models are harder to interpret because they reflect spatial and temporal filtering involved with denoising (Haufe et al., 2014; Gillis et al., 2021).

### Acoustic and lexical stimulus functions

**Figure 2.**
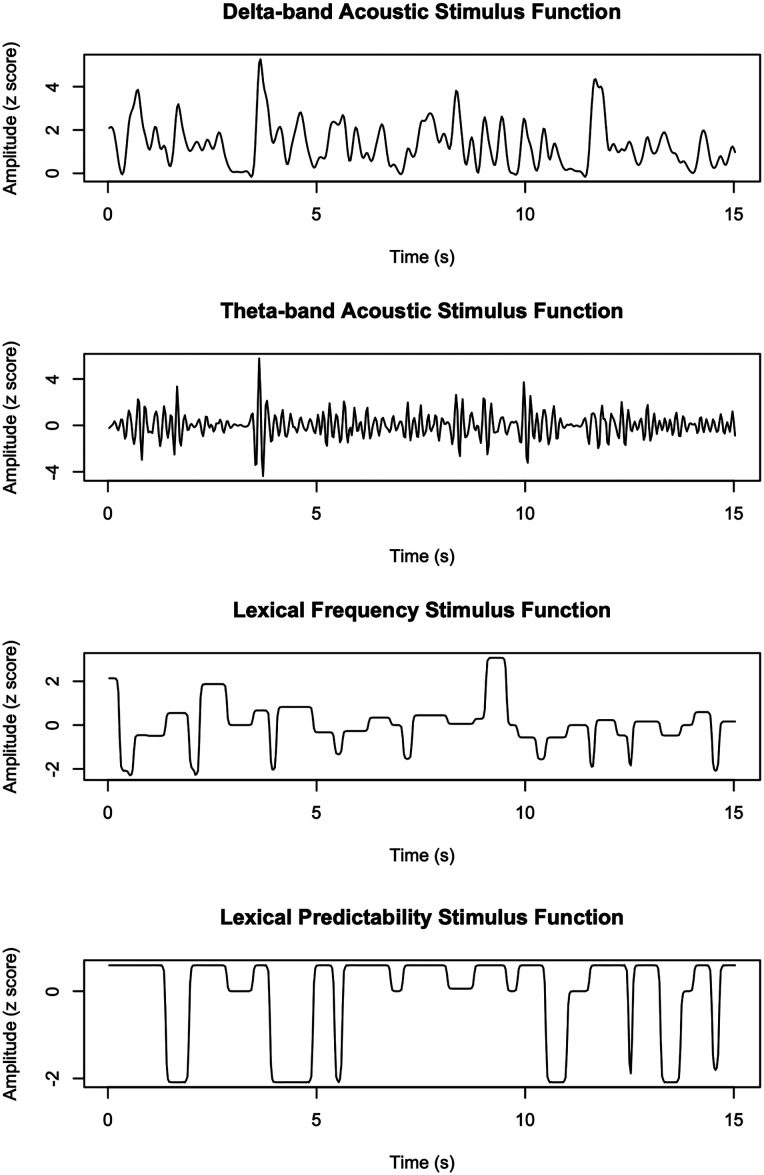
Example acoustic and lexical stimulus functions, from a 15 s section of a story spoken by one talker. The delta-band acoustic stimulus functions reproduced slower changes in the amplitude envelope (<4 Hz), roughly tracking the word rate. The theta-band acoustic stimulus functions reproduced faster changes in the amplitude envelope (4–8 Hz), roughly tracking the phoneme rate. The lexical frequency stimulus functions were higher for lower-frequency words, at zero for pauses between words, and negative for higher-frequency words. The lexical predictability stimulus functions were higher for words that were less predictable (i.e., was not predicted by ChatGPT-4 within 10 guesses), at zero for pauses between words, and lower for words that were more predictable.

The neural data was fit back to functions based on the acoustic and lexical properties of the target and distractor speech. The acoustic stimulus functions were envelopes of the separated recordings for the target and distractor talkers, calculated using a Hilbert transform. Some researchers have argued that the tracking of delta-range frequency modulations (1–4 Hz) relate to words and phrases and better reflect comprehension than the theta-range modulations that occur closer to the syllable rate (4–8 Hz; Etard and Richenbach, 2019). Thus, we created two envelopes: one low-pass filtered at 4 Hz (delta) and one high-pass filtered at 4 Hz and low-pass filtered at 8 Hz (theta), using first-order zero-phase Butterworth filters. The delta-band envelope was not high-pass filtered because, as shown in Figure 1, there was very little energy in the lowest-frequency modulations. Example acoustic and lexical stimulus functions are displayed in Figure 2.

For the lexical stimulus functions, a forced-alignment technique was used to align the words in each article to the speech recordings, using the HUBERT_ASR_LARGE pipeline within PyTorch (Hsu et al., 2021). This technique inserts word boundaries even when there are no acoustic boundaries between words, so an additional processing step removed word-boundary intervals of <250 ms; longer word-boundary intervals were retained as they typically reflected genuine pauses between words.

In order to assess lexical processing independent of the surrounding context, stimulus functions based on log lexical frequency were calculated using look-up tables from the Python wordfreq library (Brysbaert and New, 2009; van Heuven et al., 2014; Speer, 2022). We constructed novel continuous differential stimulus functions, in which regions with no speech were at zero, lexical predictors occurred through each word's duration, and the lexical predictors were centered around a duration-weighted average such that words with harder predicted lexical processing (i.e., low frequency) had positive values and those with easier predicted lexical processing (i.e., high frequency) had negative values. These differential functions were designed to find components that contrasted high and low lexical frequency rather than measuring the overall magnitude of lexical processing or acoustic onsets. Moreover, they were continuous functions, rather than discrete peaks at the onset of words, to better allow for decoding models (Crosse et al., 2021).

Our primary lexical prediction model was estimated using the GPT-4 Chat Completions API (gpt-4-0613). The prompt was “Give me 10 guesses for the next word in the following text,” and the function was given up to 100 words of the preceding text in the story (i.e., most words in the story had 100 context words, except for the early words in the story that had <100 preceding words). This function call delivered 10 unique words in order of their likelihood of completing the text, and the function call was repeated when guesses in the wrong format were received. Guesses were defined to match if they were an exact match following deletion of punctuation and conversion to lower case. Words with contractions required only a match up to the apostrophe (e.g., “he’d” and “he” were defined as a match), and multiword or hyphenated guesses that could not be resolved by repeated API calls were matched based on the first word (e.g., “and” matched the guess “and white”). Matches were recorded in terms of their serial order within the 10 guesses (i.e., positions 1–10) with a score of 11 given for words that had no matching guesses. GPT-4 offers little control over the guesses, such that there is no guarantee that the ranking of the words would be linked to their probability. However, we found that guessed words early in the list were indeed most accurate, with the percentage of matches for positions 1–10, respectively, being 31, 6, 3, 2, 2, 1, 1, 1, 1, and 1%; 51% of the words were not matched. Contrastive continuous stimulus functions were calculated as described for lexical frequency.

For comparison, we used an *N*-gram model to assess lexical predictability similar to previous studies (Koskinen et al., 2020; Broderick et al., 2021; Gillis et al., 2021). This was calculated using a five-gram model within the Google Books Ngram Viewer (English books after 1950). The predictability of each word was defined as the occurrence frequency of the *n*-gram (i.e., each word and its preceding four words of context) divided by the summed occurrence frequency of all *n*-grams with the same preceding four words of context. Stimulus functions were constructed using the log10 transform of the *n*-gram probability, with minimum probabilities of 0.001 so that *n*-grams with zero probability didn't become infinite. For further comparison, we constructed a version of GPT-4, as described above except with a maximum of five words of context, such that we could evaluate predictions using this large language model but based on local context more similar to that used in *n*-gram models. We also generated predictions using an earlier version, GPT-3 (text-curie-001), using the full preceding context. The GPT-3 API gives direct access to the estimated probabilities of words within the model, so these model probabilities were used for each target word rather than the 10-word predictions used for the GPT-4 models.

### Experimental design and statistical analysis

Pearson correlations between the original stimulus functions and the extracted neural components were used to assess the strength of neural tracking. These values were calculated separately for each article (i.e., 16 articles) and subject (*N* = 53). They were analyzed with mixed-effects models using the lme4 package (Bates et al., 2015) in R. Nonsignificant nested factors were dropped using model comparison, and the CAR package (Fox and Weisberg, 2019) used to calculate significance for the factors in the final model. The models had random intercepts for subject and stimuli; stimuli were dropped as a random factor in the lexical frequency analysis to address a singularity problem. The fixed factors were attention (i.e., target or distractor), language background (i.e., English or Spanish), and task (i.e., no secondary task or a visual *n*-back task).

In addition, permutation analyses were used to evaluate chance performance at a group level for each measure (i.e., delta and theta amplitude envelope; lexical frequency and predictability). That is, the obtained performance was compared with 100 simulations with randomly selected stimulus functions (i.e., random segments from other times in the experiment). For each simulation, a new random pairing was calculated for each individual subject, then the full analysis was run on these random pairings, producing an average correlation across subjects for each of the 100 randomizations. In every case, the obtained average correlations exceeded 100% of the random simulations, indicating that auditory and lexical tracking were greater than chance at the group level.

## Results

The behavioral task was primarily designed as an incentive for subjects to listen to the stories; there were two questions after each article, on specific facts within the articles (e.g., “Where were the author's parents born?” Answer: New Jersey). A logistic mixed model analysis revealed that native English speakers (mean proportion correct = 0.62) were more accurate than native Spanish speakers (mean proportion correct = 0.39), *χ*^2^_(1)_ = 14.13, *p *< 0.001, but there was no significant effect of the visual task or an interaction between these two factors, *p* > 0.05.

**Figure 3.**
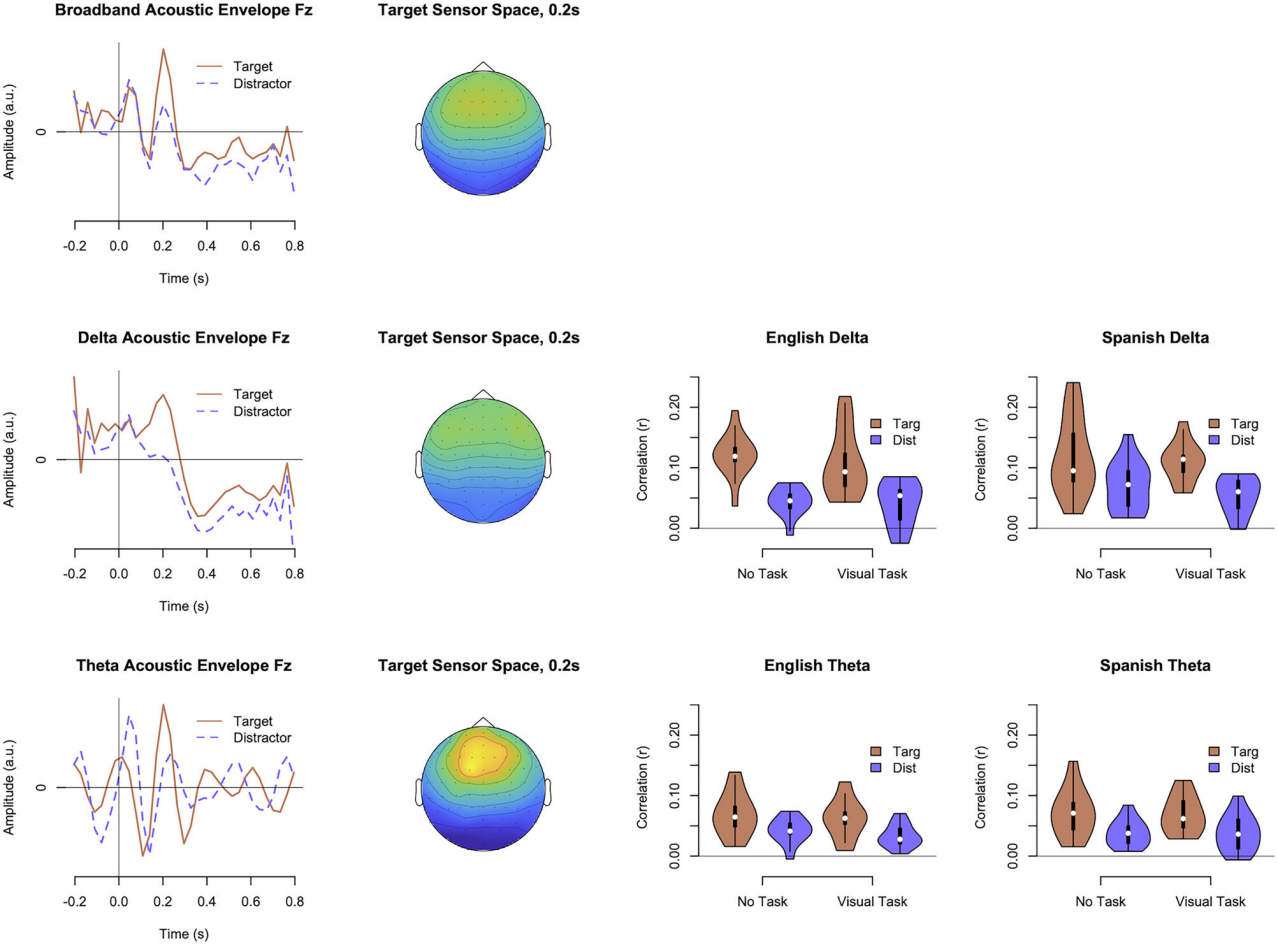
mTRF components and sensor spaces for a broadband acoustic envelope of the speech signal (<8 Hz), a delta-band acoustic envelope (0–4 Hz), and a theta-band acoustic envelope (4–8 Hz). The components largely had the temporal structure of a P1-N1-P2 auditory ERP, except that the delta and theta components also acted as time-domain filters to match the neural signal to the frequency content of the envelopes. Violin plots for the delta and theta bands display the strength of neural tracking as a function of talker attention (Targ vs Dist), language groups, and task.

Figure 3 displays the mTRF components for acoustic tracking. For reference, we first calculated tracking for a broader amplitude envelope (0–8 Hz), which resembled an auditory evoked response potential, with P1-N1-P2 peaks and stronger responses for frontal-central electrodes; this is similar to what has been previously found (Crosse et al., 2016). The components for the delta band were related to the broader-band components, with a peak at P2 combined with a time-domain low-pass filter (i.e., attenuating higher-frequency components of the neural signal to better match the delta-band auditory envelopes). The components for theta similarly tracked the P1-N1-P2 peaks combined with a theta-band time-domain filter and had a more centrally concentrated sensor space. The violin plots in Figure 3 display how strongly the decoded neural components correlated with the original stimulus functions, with a higher correlation indicating stronger neural tracking. Within a mixed-model analysis of these correlations for delta, there was a main effect of attention, with stronger tracking for target talkers than distractors, *χ*^2^_(1)_ = 477.98, *p *< 0.001, and an interaction of attention and language, *χ*^2^_(1)_ = 11.29, *p *< 0.001, with less difference between target and distractor talkers for the Spanish speakers. For theta, there was only a main effect of attention, *χ*^2^_(1)_ = 254.84, *p *< 0.001. There were no other significant main effects or interactions, *p *> 0.05.

**Figure 4.**
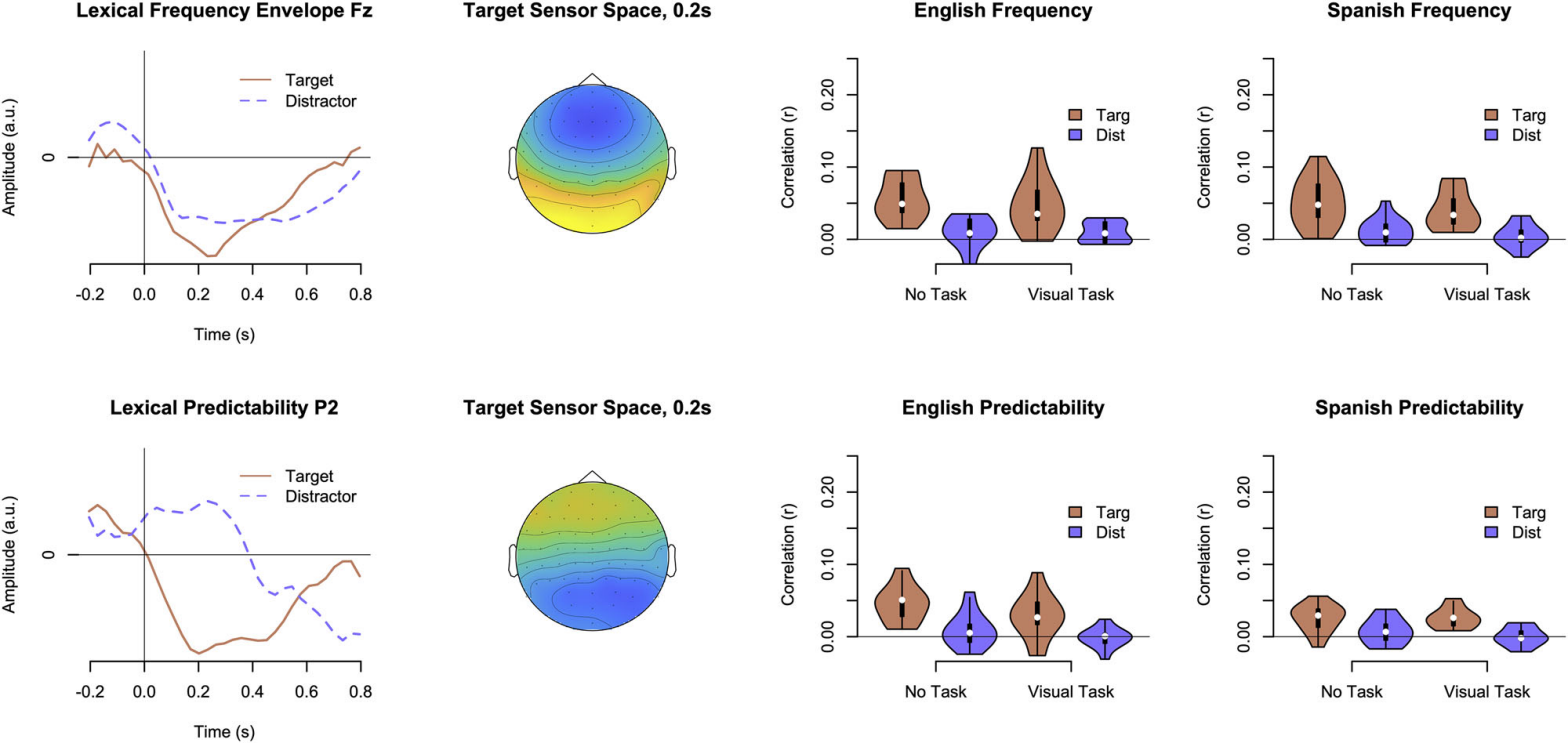
mTRF components and sensor spaces for stimulus envelopes that varied lexical frequency and predictability, with frequency having a more frontal distribution and prediction being more parietal. Violin plots for lexical frequency and predictability display the strength of neural tracking as a function of talker attention (Targ vs Dist), language groups, and task.

Figure 4 displays mTRF functions for lexical frequency and predictability (GPT-4). We were able to extract a separate component for lexical frequency, with a broad negative component for the target peaking 233 ms after the stimulus onset and with frontal scalp distribution. The coefficients somewhat resembled an auditory N400 ERP, except that the strongest components were more frontal than previously found for lexical frequency (Winsler et al., 2018; Gillis et al., 2021). The peak was earlier than found in typical N400 experiments (i.e., negative peak at 400 ms), but comparing time scales is not straightforward given that our contrastive stimulus functions were not solely driven by stimulus onsets. A mixed model analysis revealed that there was only a significant effect of attention, *χ*^2^_(1)_ = 269.30, *p *= 0.001, with stronger tracking for the target talker than the distractor; there were no other significant main effects or interactions, *p *> 0.05.

The GPT-4 lexical predictability model also produced distinct mTRF components, with a broader time course than for lexical frequency (e.g., greater overall latency) and with a more parietal scalp distribution (Fig. 4); this is a clearer difference than found previously for lexical frequency and predictability using *n*-gram models (Gillis et al., 2021). Within a mixed-model analysis of these correlations, there was a main effect of attention, with stronger tracking for target talkers than distractors, *χ*^2^_(1)_ = 155.88, *p *< 0.001, and a significant interaction between language background and attended speech, with a stronger separation between target and distractor talkers for the native listeners, *χ*^2^_(1)_ = 6.34, *p *= 0.011. There was a main effect of task, with correlations being lower overall when listeners completed a visual *n*-back task, *χ*^2^_(1)_ = 4.36, *p *= 0.036. There were no other significant effects or interactions, *p *> 0.05.

We compared the lexical predictions of GPT-4 with those of the older *n*-gram model, as well as alternative formulations of GPT models. One major difference between these stimulus functions is the accuracy of the predictions, although the methods to estimate word probabilities vary between methods. GPT-4 predicted 31% of words with the top guess and 49% of words with 1 of the 10 guesses. For the *n*-gram model, only 6% of the words were predicted with a >50% proportion of the relevant *n*-grams and 12% of the words predicted with a >10% proportion of the relevant *n*-grams. We also constructed GPT-4 predictions using only five words of context, such that we could use the more modern large-language model predictions but with an amount of context similar to previous *n*-gram models. Reducing the amount of context decreased the number of predictable words: 16% of the words were predicted with the top guess and 26% of the words in the top 10 guesses, or a bit more than half the predicted words of the full model. We likewise constructed predictions using GPT-3: 20% of the words were predicted with a >50% model probability and 42% of the words with a >10% model probability. It is difficult to directly compare rank predictions with *n*-gram probabilities and internal model probabilities, but the overall conclusion is that newer models using more words of the preceding context are able to better predict the words in our stories.

**Figure 5.**
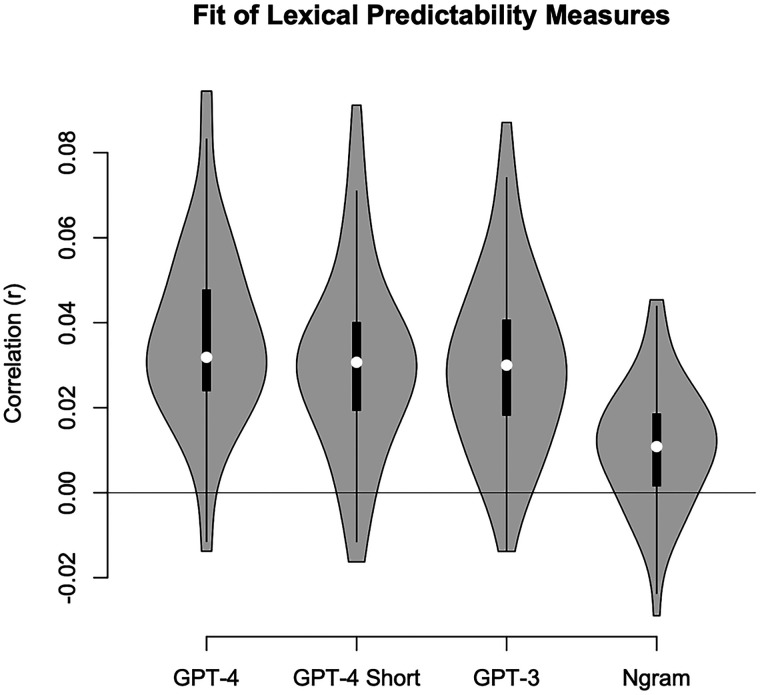
Violin plots display the strength of neural tracking across methods that estimate the lexical predictability of words: GPT-4 with up to 100 words of context, GPT-4 with only five words of context, GPT-3 with full context, and an *n*-gram model. There were significantly greater correlations for the 100-word GPT-4 predictions, suggesting that more modern models and a fuller context better account for neural data.

In order to test whether lexical tracking differed for these models, we compared the correlations between mTRF predictions and stimulus functions within a mixed model, for target talkers and not considering language and task. There was a significant main effect of measure, *χ*^2^_(1)_ = 79.44, *p *< 0.001. As displayed in Figure 5, the *n*-gram models were the worst overall, with all GPT models being of similar magnitude. That being said, paired *t* tests revealed that the GPT-4 model with 100 words of context had greater correlations with the neural data compared with GPT-3 with the full word context, *t*_(52)_ = 2.85, *p *= 0.006, GPT-4 with only five words of context, *t*_(52)_ = 3.08, *p *= 0.003, and the *n*-gram model, *t*_(52)_ = 6.94, *p *< 0.001. Thus, the latest advances in large language models are an improvement in terms of the numbers of words predicted and the fit to the neural data, although a range of GPT models offer similar views of the neural data.

A key aim of this study was to investigate whether lexical predictions were coupled to differences in auditory tracking. Previous work (Broderick et al., 2019) suggested that auditory tracking was greater for words that were more semantically related to the previous context and that this effect was strongest in the first 100 ms of each word. Also, delta-band auditory tracking has been found to be related to comprehensibility (i.e., whether the speech was in a language the listener knew or not; Etard and Reichenbach, 2019). This effect was found 100 ms preceding the stimulus, suggesting a possible role of predictive lexical processing although it is also possible that this was caused by temporal smearing within the analysis. Signal quality (i.e., amount of added noise) was more related to theta-band auditory tracking. In our present analysis, we examined whether auditory tracking varied with lexical predictability and frequency. Auditory tracking was measured over the first and second halves of each word in the stories produced by target talkers, with the words split in half to introduce a time element related to previous work (Broderick et al., 2019). Within a mixed-model analysis, these correlations were the dependent factor, which were predicted by fixed factors of GPT-4 lexical predictability, lexical frequency, half (first or second half of each word), task, and the native language of the listener, with word duration added as a covariate as a control for the acoustic clarity of each word.

**Figure 6.**
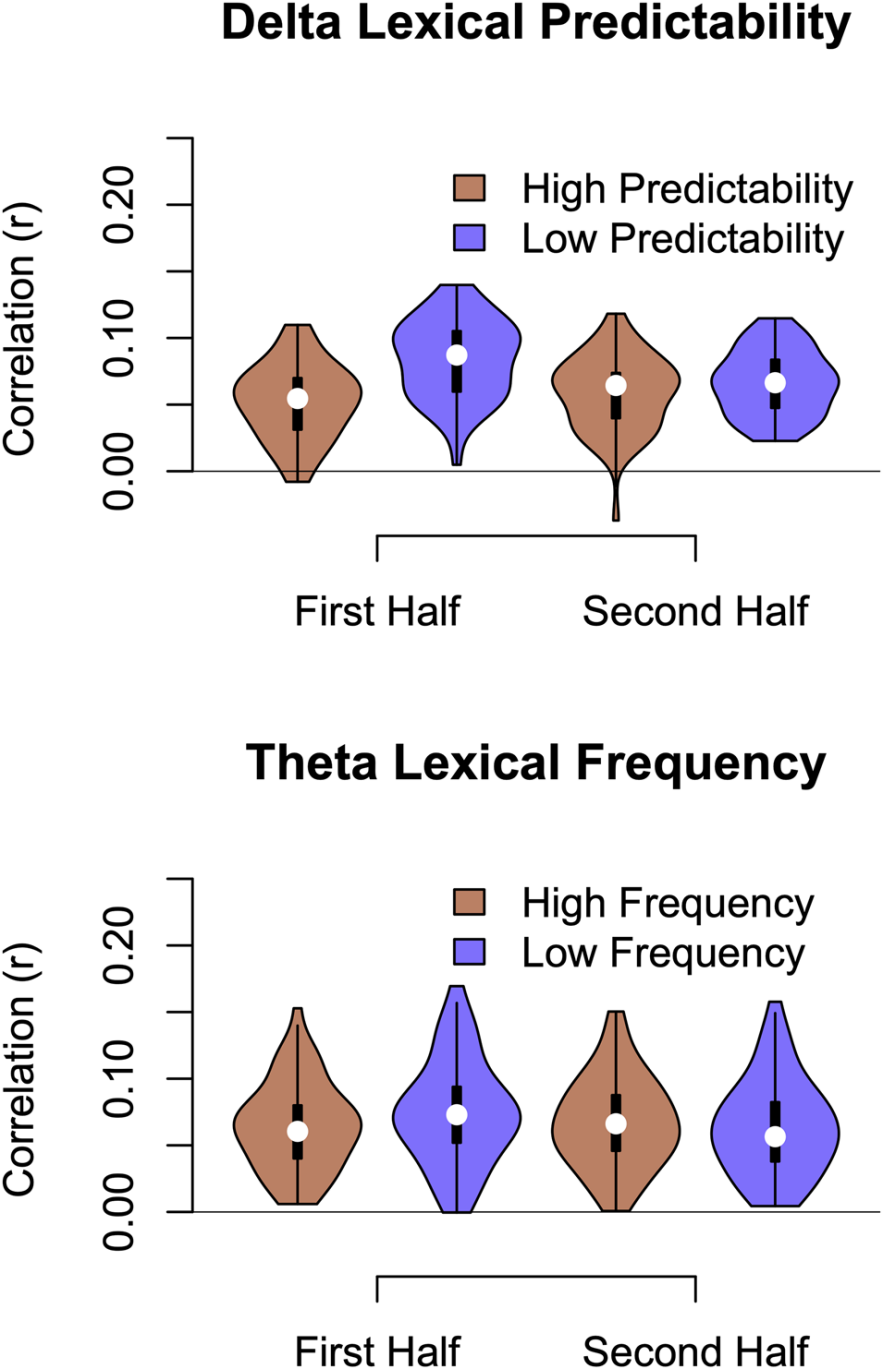
Violin plots display the main findings for how lexical predictability and frequency affect auditory tracking. Both lexical measures were split at the median for display, even though they were entered into the analyses as continuous factors. Delta-band auditory tracking was greater for less lexically predictable words, particularly during the first half of the words. Theta-band auditory tracking was similarly affected by lexical frequency, although not to as strong an extent; during the first half of each word, words with lower lexical frequency had greater auditory tracking.

Mixed-model analyses on delta-band auditory tracking revealed a significant main effect of lexical predictability, *χ*^2^_(1)_ = 57.65, *p *< 0.001, with greater auditory tracking for words that were less predictable (mean correlations of 0.075 and 0.056, respectively, for words that were not predicted by Chat-GPT and words that appeared in the first 10 predictions; Fig. 6). There was also a main effect of half, *χ*^2^_(1)_ = 6.49, *p *= 0.010, with greater auditory tracking over the first half of the word than the second half (respective mean correlations of 0.069 and 0.063). There was an interaction of lexical predictability and half, *χ*^2^_(1)_ = 43.40, *p *< 0.001, with the tracking difference for low and high predictability words over the first half (respectively, 0.084 and 0.052) being greater than the low-high predictability difference over the second half (respectively, 0.066 and 0.059). Previous work, with easier listening conditions, had shown greater correlations with more comprehensible words, and those that were semantically related to the previous context (Broderick et al., 2019; Etard and Reichenbach, 2019). Under our more difficult conditions, we are more finding an effect of effort modulated by lexical factors, with greater auditory tracking for words that are less predictable from the context, particularly in the early parts of the words where the word is less identifiable because less acoustic information has been heard. Finally, there was a main effect of duration, *χ*^2^_(1)_ = 5.30, *p *= 0.021, with shorter words having lower correlations (mean, 0.060) than longer words (mean, 0.066). There were no other significant effects, including those involving lexical frequency, *p *> 0.05.

Parallel analyses with theta-band auditory tracking revealed similar results, although with greater lexical frequency effects. There was a significant main effect of lexical predictability, *χ*^2^_(1)_ = 16.20, *p *< 0.001, with greater auditory tracking for words that were less predictable (mean correlations of 0.071 and 0.059, respectively, for lesser and greater predictability; Fig. 6). There was a main effect of half, *χ*^2^_(1)_ = 3.98, *p *= 0.046, with greater auditory tracking over the first half of the word than the second half (respective mean correlations of 0.068 and 0.064). There was a main effect of frequency, *χ*^2^_(1)_ = 5.12, *p *= 0.024, with greater auditory tracking for low-frequency words (mean, 0.068) than high-frequency words (mean, 0.064). There was a significant interaction with frequency and half, *χ*^2^_(1)_ = 15.74, *p *< 0.001, with the auditory tracking differences for low- and high-frequency words over the first half of each word (respective means 0.073 and 0.062) being greater than over the second half (respective means 0.063 and 0.065). Finally, there was a complex three-way interaction between frequency, language, and task, *χ*^2^_(1)_ = 6.80, *p *= 0.009, with the greatest frequency effect occurring under the most difficult condition (i.e., Spanish speakers performing the visual task; respective means for low and high frequency, 0.072 and 0.058).

## Discussion

The main new finding of this work is that lexical prediction and frequency couple with auditory tracking under difficult conditions, with stronger acoustic tracking of a target talker in the presence of a competing talker when words are less predictable and lower in frequency. This result may seem at odds with previous work demonstrating that more accurate auditory tracking is associated with higher intelligibility and that auditory tracking can be greater, in quiet conditions, for words that are semantically related to the previous context (Gross et al., 2013; Peelle et al., 2013; Ding and Simon, 2014; Broderick et al., 2019; Etard and Reichenbach, 2019). However, auditory tracking can also be affected by listening effort, which often has a U-shaped function (Davis and Johnsrude, 2003; Ohlenforst et al., 2017). That is, enhancements of auditory tracking can be found under moderately difficult listening conditions that require greater attention to the signal, such as signal degradation or difficult accents (Song and Iverson, 2018; Song et al., 2020; Hauswald et al., 2022), but greater difficulty can then reduce auditory tracking (Petersen et al., 2017; Hauswald et al., 2022). Related momentary changes in listening effort have been found in pupillometry studies, with increases in pupil size occurring in response to a mispronounced target (Winn, 2023).

Our conclusion here is that we are observing an adaptation that increases the signal-to-noise ratio at times when an accurate acoustic representation is more needed. It is unlikely that this modulation of attention happens on a conscious level, as this would require continuous changes in listening effort at less than word-level durations (generally less than half a second) during the course of an hour-long EEG recording session. However, this kind of rapid modulation of processing is exactly what is thought to happen automatically at a lexical level during speech recognition (Brown and Hagoort, 1993; Kutas and Federmeier, 2000; Federmeier, 2007), which makes it seem plausible that predictions within lexical processing are directly feeding down to affect auditory processing. That being said, the direction of these effects are hard to prove. For example, we have shown effects of lexical frequency on theta-band tracking, and lexical frequency is related to difficulty of lexical access rather than prediction. Other work (Etard and Reichenbach, 2019) has suggested that their apparent predictive modulation of delta-band tracking may not be completely definitive because time was blurred in their analyses. Likewise, in a correlational study like ours, with continuous read stories, this apparent effect could be caused by some uncontrolled factor (e.g., possible differences in the way speakers produce predictable and unpredictable words). But there is converging evidence that auditory tracking and lexical prediction are coupled, rather than only being a feedforward process of auditory processing projecting onto a lexical processing level.

The form-based representation of words in the lexicon is generally thought to be phonetically detailed, with graded phonetic and talker-specific information activating lexical representations (Goldinger, 1998). It has thus been argued that it is better for continuous multidimensional phonetic information to be fully available at a lexical decision stage, rather than have top-down processes that narrow or bias the information at lower levels of processing (Norris and McQueen, 2008). These hypotheses may seem contrary to the top-down mechanism proposed here, but the critical element is background noise. That is, the lexical information from a competing talker is a source of interference rather than of information, and it may indeed be beneficial for the speech processing system to attenuate the neural signals from unattended talkers at as low a level as possible (Dai et al., 2022). It thus appears that lexical processing can have top-down effects under circumstances where discarding information has a functional value.

It was somewhat surprising that nonnative listeners had less difference in tracking for target and distractor talkers, compared with native speakers, particularly for auditory tracking. Previous work found that non-native speakers have enhancements for tracking because of greater listening effort and that native speakers can have enhanced tracking for non-native accents (Song and Iverson, 2018; Song et al., 2020). This difference could be due to the U-shaped listening effort function (Davis and Johnsrude, 2003; Ohlenforst et al., 2017); the listening condition used here may have been difficult enough such that non-native listeners had reduced tracking, rather than being in a zone of more moderate difficulty that could be compensated for by additional effort. The effect of language background was not uniform across our measures, being nonsignificant for lexical frequency. This opens up the possibility that more basic aspects of lexical processing may be more uniform across listeners compared with more complex lexical–semantic prediction, but such a possibility requires further research.

Overall, the results demonstrate that lexical processing can be broken down into separable components, a finding known from previous work (Winsler et al., 2018) but demonstrated here on continuous data with large-language model estimates of lexical predictability. With these new models, it is now clear that prediction is not a rare phenomenon for frequent combinations of words or contrived test materials with highly predictable words; nearly half of the words in our articles could be predicted by GPT-4. Moreover, it appears that these more frequent predictions better model neural data than do older models or predictions based on less context. The current lexical predictions appear to use information from the GPT-4 training set that exceeds normal listener knowledge. GPT-4 can, for example, predict the names of ski resorts in Argentina, obscure book titles from author names, and biographic details of lesser-known celebrities. Large language models may reach a point in which their predictions are no longer useful for assessing neural processing. It is likely that open-source language models, retrained through a collaboration of natural-language-processing researchers and speech neuroscientists, may be required to make further progress on understanding lexical processing under realistic conditions.

## Synthesis

Reviewing Editor: Anne Keitel, University of Dundee Decisions are customarily a result of the Reviewing Editor and the peer reviewers coming together and discussing their recommendations until a consensus is reached. When revisions are invited, a fact-based synthesis statement explaining their decision and outlining what is needed to prepare a revision will be listed below. The following reviewer(s) agreed to reveal their identity: Anne Kösem, Lars Hausfeld. Note: If this manuscript was transferred from JNeurosci and a decision was made to accept the manuscript without peer review, a brief statement to this effect will instead be what is listed below.

Both reviewers and the editor agreed that this is an interesting study and that the response to previous reviews have improved the manuscript. However, the reviewers have a few remaining comments. These can be summarised as follows:

1) More methodological details are necessary (see below).

2) A couple of control analyses have been suggested regarding different delays, forward models, and additional predictors.

3) Two more figures (one for methods and one for results) would support the clarity of the methods and findings.

4) Please add behavioural scores.

Please see below for the unabridged reviewer comments, which detail these issues as well as other issues. Please respond to each of the comments in a point-by-point manner.

*****

Reviewer #1

*Advances the Field (Required)

In this EEG study, individuals were instructed to actively listen to one specific speech while disregarding any other distracting speech. The researchers employed machine learning techniques to examine how the brain tracks and processes the distractor and target speech. They investigated how this tracking is influenced by factors such as the amplitude of the acoustic envelope, the frequency of the words, and the predictability of the words, which were estimated using GPT-4 and n-grams models. The study included both native and non-native English speakers who participated in either an isolated auditory task or a combined auditory and visual task. The results of the study revealed a distinct difference in the ability to track the target speech compared to the distractor speech among non-native listeners. Additionally, the researchers observed an interesting interaction between lexical predictability and auditory processing. They found that the acoustic tracking in the delta frequency range was weaker for words that were more predictable.

*Software Comments

Unable to test (no code available)

*Comments to the Authors (Required)

I thank the authors for their extensive revisions, which have greatly improved the clarity of the paper. However, I still have some comments regarding the description of the main findings and their interpretation. The key finding is discussed on pages 290-329, but there is a need for more specific information regarding the methods used and the details of the analysis.

1. I would suggest explicitly describing the auditory tracking measure used for this analysis in the methods section. It is unclear to me what duration of segments was used to compute this measure. Is it based on word-duration level or on longer segments?

2. Based on my understanding, the correlation was performed over the full word segments without considering different time windows. As suggested by reviewer 2 in the previous review, it would be worthwhile to investigate the impact of lexical predictability on acoustic tracking over different delay windows (e.g., -250ms to 750ms according to word onsets). Previous findings have shown that the enhancement of acoustic tracking due to semantic context is strongest in the first 100 ms of a word's utterance (Broderick, M. P., A. J. Anderson and E. C. Lalor (2019). "Semantic Context Enhances the Early Auditory Encoding of Natural Speech." Journal of Neuroscience).

3. It would be beneficial to include a figure that visually represents the main findings. This would help to emphasize their importance.

4. Importantly, it is crucial to ensure that any observed effects of semantic predictability on acoustic tracking are not solely attributable to changes in the speech acoustics, but rather reflective of genuine top-down neural effects. Previous studies have shown that semantic predictability is associated with differences in word pronunciation, such as envelope variability, average relative pitch, and average resolvability (Broderick et al., 2019). It would be interesting to account for these acoustic confounds by regressing them out, as conducted in previous research (Broderick et al., 2019).

*****

Reviewer #2

*Advances the Field (Required)

main advancement is methodological (applying GPT4 to continuous speech stimuli), conceptually the idea/observation that predictability might affects acoustic tracking of continuous speech is intriguing.

*Statistics

the analyses and stimulus functions are not 100% clear (see reply).

*Comments to the Authors (Required)

This is a resubmission of a previously reviewed manuscript. The authors addressed most of the points, added, among other information, the comparison of GPT3 and the analysis of delta and theta bands.

Please find below my remaining points

1. The behavioral scores, even though they might not be showing differences between tasks/listeners, are still important to report for both transparency as well as interpretation. Non-significant differences as well as the performance level provide a good estimation of the general difficulty. Not finding behavioral differences in presence of neural difference is an interesting finding itself that should be commented upon.

2. The authors didn't fully address the point of forward models raised in the previous review. One main advantage of forward models is the inclusion of more than a single stimulus function, thus addressing shared variance between descriptors/functions. For example, acoustics and GPT probabilities will show some correlation as the word onset will be lead to acoustic and as well a GPT/word-based deflection of the stimulus function. One can for example test whether adding GPT information will add to n-gram probabilities (i.e. is the model performance for n-gram or n-gram + GPT4) in terms of model performance (e.g. Di Liberto et al., 2015).

3. It is unclear how permutations were performed. While I welcome that these have been done, it is necessary to provide how exactly these were done. For example, the authors write in the reply that permutations showed p<.001 while in the text it is mentioned that 100 permutations were performed (only providing p<.01 as lowest significance). In addition, it is unclear whether permutation are performed on the single-participant level (and then later on the group level) or 'somehow' immediately on the group level.

4. It's unclear why scores for native Spanish speakers are unnecessary. Are the current language performance tests not sensitive enough that differences between native English and non-native but highly proficient speakers can be found? Please add this information to the manuscript as the difference between native/non-native is one of the main results.

5. Regarding the reply to R2-point 8, the type of analysis is not affected by the stimulus function as suggested as I understand the analysis. Currently, the author use -250-750ms as a window, restricting it to an early and late window could indicate the timing of the effect, while the delay-specific analysis would show the temporal development of the effect.

6. Please add a figure regarding the different stimulus functions. This would help readers in following the performed analyses and interpret results.

7. As indicated during the review process, the n-gram and GPT4-based results are difficult to compare; given the output of GPT3 and the specific n-gram model, these are more readily comparable. Comparing GPT4 models between each other are valid as well. However, comparing GPT4 with n-gram performance based on different information is difficult. Thus, presenting the performances as Figure 4 does might be misleading if this is not clearly indicated in the figure, caption and text.

8. I did not find information about the EEG data used for Figure 3. Before the data was analysed in different frequency bands, has this been done here as well? If so, what type of frequency band is presented, if not, please explain why this hasn't been done for this analysis.

9. TRFs in Figures 2 and 3 do not show the standard error, please add those.

10. The optimization regarding the regularization parameter for the mTRF analysis is not described (range of values, how was it selected). Please report how these were set or please mention if there was no regularization performed.

11. L.224: the broadband analysis indicates 0-8Hz while the data were high-pass filtered at 0.1 Hz, please change this.

12. The data and stimulus functions are interesting for the community, it is unclear whether data and code will be shared according to standard OpenScience practices
